# Management of syphilitic hepatitis

**DOI:** 10.1186/s12876-020-01496-5

**Published:** 2020-11-12

**Authors:** Abdurrahman Kaya, Sibel Yıldız Kaya

**Affiliations:** 1 Department of Infectious Diseases, İstanbul Training and Research Hospital, Istanbul, Turkey; 2 Infectious Diseases Unit, Sungurlu State Hospital, Çorum, Turkey

**Keywords:** Syphilis, Rash, Liver enzyme

## Abstract

Syphilis is a sexuality transmitted disease caused by *Treponema pallidum*. Liver involvement is very rarely seen and occurs in the second phase of the disease. Syphilitic hepatitis generally is mild clinical condition and is characterized by high serum alkaline phosphatase level, often with normal or only slightly abnormal transaminases. The skin eruptions are classically diffuse, symmetric maculopapular rashes involving trunk and extremities. Involvement of palms and soles is a strong clue to the diagnosis of secondary syphilis. Therefore, syphilitic hepatitis should be included in the early differential diagnosis in patient with abnormal liver enzyme, especially increased alkaline phosphatase, and rashes involving palms and soles.

**Dear editor,**

We have read with great interest the manuscript entitled *Syphilitic hepatitis: a case report and review of the literature* [1]. It presented a case of syphilitic hepatitis (SH) that had jaundice, rashes and abnormal hepatic enzyme level. However, we have some concern regarding the management of the patient.

## Discussion

The diagnosis of the patient is clear and was confirmed with the combination of serology and liver biopsy but dermatology consultation was not performed despite unresolved rashes. SH is the component of the secondary syphilis that bacteria disseminate all over the body. It is characterized by high serum alkaline phosphatase level, often with normal or only slightly abnormal transaminases. In patients infected with human immunodeficiency virus, about 50% present with mixed enzyme pattern and in 13% only hepatocellular enzymes are elevated. The skin eruptions are classically diffuse, symmetric maculopapular rashes involving trunk and extremities. Involvement of palms and soles is a strong clue to the diagnosis of secondary syphilis [2, 3]. In this case, the palms and soles were also involved. Therefore, dermatology consultation could provide early diagnosis and prevent unnecessary invasive procedure such as liver biopsy. We thought liver biopsy was early performed.

Secondly, the authors performed genital examination on admission. However, after diagnosing syphilis, they did not state anything about his sexual history including time to unprotected sex and presence or absent of previous genital lesion such as chancre. Therefore, the detailed sexual history of the patient should have been taken.

Thirdly, a single dose of penicillin G benzathine (2.4 million units intramuscularly) is the standard regimen for early syphilis (primary, secondary and early latent). Generally, SH is a mild condition and well responds to the treatment [3–5]. No clinical data is available for more prolonged treatment. However, in this case, the patient was given penicillin G per week for successive 2 months.

## Conclusion

SH is very rare condition that was often overlooked and seen in secondary stage of the disease. A single dose of penicillin G benzathine is the standart treatment for SH. SH should be included in the early differential diagnosis in patient with abnormal liver enzyme, especially increased alkaline phosphatase, and rashes involving palms and soles.

## Data Availability

Not applicable.

## Abbreviation

SH: Syphilitic hepatitis
